# Decolorization of Azo and Anthraquinone Dyes Using Recombinant Horseradish Peroxidase A2A Isoenzyme Produced by *Komagataella phaffii*

**DOI:** 10.1007/s12010-025-05239-8

**Published:** 2025-05-03

**Authors:** Nurgul Abul, Seyda Yildiz Arslan, Yagmur Unver, Hasan Ozdemir

**Affiliations:** 1 Department of Chemistry, Institute of Science and Technology, Atatürk University, Erzurum, Türkiye; 2 Department of Molecular Biology and Genetics, Institute of Science and Technology, Atatürk University, Erzurum, Türkiye; 3 Department of Molecular Biology and Genetics, Faculty of Science, Atatürk University, Erzurum, Türkiye; 4 East Anatolia High Technology Application and Research Center (DAYTAM), Atatürk University, Erzurum, Türkiye; 5 Department of Chemistry, Faculty of Science, Atatürk University, Erzurum, Türkiye

**Keywords:** Recombinant HRP A2A isoenzyme, Azo dye, Anthraquinone dye, Decolorization, Water pollution

## Abstract

Water pollution is a significant issue due to industrialization and population growth, and one of the main sources of wastewater is synthetic dyes. The textile sector is particularly affected by dyes like azo and anthraquinone dyes, which are difficult to degrade and produce toxic organic waste. Currently, synthetic dyes are processed through physical and chemical methods, which have financial and methodological disadvantages. Horseradish peroxidase (HRP) is a widely studied enzyme for purifying pollutants like dyes and phenols in wastewater. However, their high cost makes them a costly option. Recombinant protein production is suitable for the mass production of stable and resistant enzymes. In this study, the decolorization potential of recombinant HRP A2A (rHRP A2A) isoenzyme secreted by *Komagataella phaffii* and purified by affinity technique in a single step on Acid blue 113, Alizarin red, and Remazol brilliant blue R was presented for the first time, and the optimal conditions for the highest decolorization rate were determined. Fe^2+^ and Mn^2+^ metal ions increased enzyme activity by 158.62% and 79.54%, respectively. Color removal with 0.006 EU/mL rHRP A2A for Acid blue 113, Alizarin red, and Remazol brilliant blue R was observed at 71.27, 62.26, and 31.22%, respectively. ABTS served as a redox mediator, significantly increasing the rate of dye decolorization in a shorter period at the specified concentration.

## Introduction

Increasing water pollution due to industrialization and rapid population growth has been a major concern in the last century. The discharge of large quantities of industrial waste contaminated with synthetic dyes into natural water reservoirs is one of the leading causes of water pollution [1]. Synthetic dyes are commonly utilized across various industrial sectors such as chemical, biomedical, pharmaceutical, food, textile, leather, printing, and plastics [1]. It is estimated that their production can reach 7 × 10^5^ tons per year, and the industries use 10,000 types of pigments and dyes annually [2].

Over the past 20 years, environmental problems related to the textile sector have drawn much attention. For the past few decades, the textile sector has been a major source of highly polluted wastewater due to its extensive use of dyes of various kinds. Up to 80% of synthetic azo dyes used in dyeing applications are thought to be produced annually by the industry. During dyeing, 10–15% of the colors are disposed in wastewater because they do not bond to fibers. Therefore, wastewater from processing plants is estimated to increase geometrically due to industrial growth and expansion [3, 4]. The primary issue arising from the release of this wastewater is water pollution. Wastewater discharge impacts the aquatic ecology and, therefore, the health of the flora and fauna [5]. These colorants threaten marine life, the environment, and human health when they contaminate water. The chemical nature of these dyes, which tends to linger in nature, has negative effects. Moreover, these dyes harm aquatic organisms’ ability to survive and alter the makeup of aquatic ecosystems [6–9].

Based on chemical structure, synthetic dyes are classified into 20–30 groups, with the majority of these dye groups being composed of azo (60–70%) and anthraquinone (15%) [10]. Azo dyes are the most widely used class of synthetic dyes in the textile industry and the most commonly found in industrial wastewater [11]. Around 90% of the azo dyes are assumed to be released into the environment without adequate treatment [12]. Because of their stability and ease of synthesis, these dyes are favored. However, the toxicity, carcinogenicity, and mutagenicity of synthetic azo dyes and/or their metabolites (aromatic amines) are well described [13]. Acid blue 113 (AB 113), an azo dye used in textile production, accounts for about 60% of the total market [14]. The anionic azo dye Acid blue 113 has aromatic sulfonic groups that cause ecological toxicity [15].

The second most significant family of dyes used extensively in the textile industry, anthraquinone dyes, are simply applied and offer a wide range of colors and shades [2]. Remazol brilliant blue R (RBBR, disodium 1-amino-9,10-dioxo-4-{3-[2-(sulfonatooxy)ethane-1-sulfonyl]anilino}−9,10-dihydroanthracene-2-sulfonate) is an anthraquinone dye mostly used in textile production. This dye is difficult to degrade and produces toxic organic waste [16]. Another anthraquinone dye, Alizarin red (AR; 3,4-dihydroxy-9,10-dioxo-9,10-dihydroanthracene-2-sulfonic acid), is a widely used dye and acid–base indicator. Approximately 10 to 15% of the dye used in the textile industry ends up in the dye effluent. AR can double the efficiency of oxidative damage by inserting between base pairs of the DNA double helix [17].

Many approaches have been used to remove dye from textile wastewater and lower overall process costs, including chemical oxidation, physicochemical techniques (adsorption, coagulation/flocculation, and reverse osmosis), microbial or electrochemical discoloration, and, most recently, the use of various enzymes [18]. In contrast to physical/chemical procedures, biological techniques have been developed to clean up many toxic pollutants in a more cost-effective, efficient, and ecologically friendly manner [19–21]. The capacity of pollutants to function in a wide range of concentrations, increased specificity, ease of use, improved standardization, and storage have all contributed to the growing interest in the enzyme degradation of dyes [22, 23]. An array of oxidoreductive enzymes, including horseradish peroxidase (HRP), several fungal lignin peroxidases (LiP), cytochrome C peroxidase, manganese peroxidase (MnP), and chloroperoxidase, are implicated in the process of dye decolorization. Together with hydrogen peroxide, these enzymes are excellent oxidant agents for degrading dyes [24–28].

Horseradish peroxidase (HRP; EC 1.11.1.7) is an oxidoreductase enzyme that contains heme and is mostly isolated from the root of the horseradish plant (*Armoracia rusticana*) [29]. It breaks down hydrogen peroxide (H_2_O_2_) to accelerate the oxidation process of various substrates. Hazardous substances such as dyes, pharmaceuticals, phenols, and xenobiotics have recently been decontaminated using HRP [30]. Numerous investigations revealed that HRP effectively cleaved aromatic azo compounds in the presence of H_2_O_2_, degraded and precipitated the azo dyes [31–34], and promoted the breakdown of anthraquinone dyes [35–37].

This study aimed to investigate the decolorization potential of recombinant HRP A2A (rHRP A2A) isoenzyme produced extracellularly by *Komagataella phaffii* (previously described as *Pichia pastoris*) and purified by affinity technique in a single step from the culture medium on Acid blue 113, Alizarin red, and Reactive blue 19 (Remazol brilliant blue R, RBBR) and to determine the optimum conditions for the highest rate of decolorization.

## Material and Methods

### Materials

**Fig. 1:**
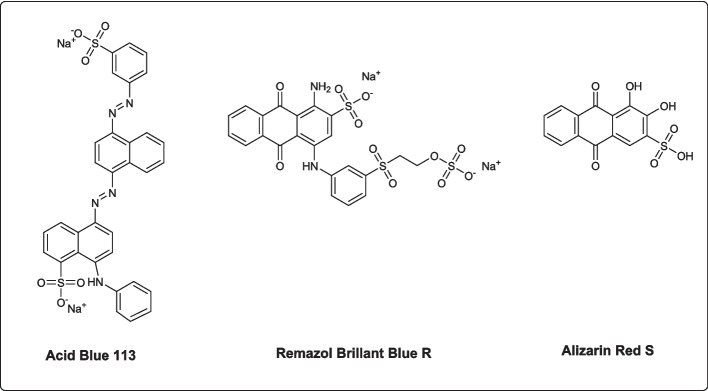
Dyes used for decolorization experiments

Acid blue 113, an azo dye, Remazol brillant blue R and Alizarin red, anthraquinone dyes (Fig. 1), o-dianisidine, 2,2′-azino-bis (3-ethylbenzothiazoline-6-sulfonic acid (ABTS), and H_2_O_2_, (hydrogen peroxide) were purchased from Sigma-Aldrich. 3-Amino 4-kloro benzohydrazide affinity column was synthesized as in our previous study [38]. Sodium hydroxide (NaOH), yeast extract, and potassium dihydrogen phosphate (KH_2_PO_4_) were obtained from Merck. Yeast extract peptone dextrose (YPD) (Sigma-Aldrich), bacteriological peptone (Neogen), yeast nitrogen base (YNB), glycerol (ChemCruz), casamino acid (USBiological), and agar (Neogen) were also used.

### Extracellular Production of Recombinant HRP A2A (rHRP A2A)

In our previous study, we successfully achieved heterologous protein expression of HRP A2A by *Komagataella phaffii* (*Pichia pastoris*) [38]. The recombinant yeast cells were grown on YPD agar at 30 °C for 48–60 h. YPD agar was prepared by dissolving 5 g of YPD and 2 g of agar in 100 mL of purified water and sterilized by autoclaving at 121 °C for 15 min. After incubation, the cells were inoculated in 12.5 mL of pre-culture (BMGY, Buffered Minimal Glycerol-Complex Medium) medium containing 1% KH_2_PO_4_ (100 mM), 1% yeast extract, 1.34% YNB, 2% peptone, 1% glycerol, and 25 μL of 500 × B (0.02% Biotin) and then incubated at 30 °C at 225 rpm until the culture reached an OD_600_ value of approximately 2. After incubation, cells were harvested from culture medium by centrifugation and resuspended in 25 mL of the production (BMMY, Buffered Minimal Methanol-Complex Medium) medium containing 1% KH_2_PO_4_ (100 mM), 1% yeast extract, 1% casamino acid, 2% peptone, 1% sorbitol, 50 μL 500X B, and 1.34% YNB to induce protein expression. Methanol was added to the production medium every 24 h to a final concentration of 0.5% for induction maintenance. The obtained culture liquid at the end of 72 h of incubation was used to load onto the affinity column.

### Purification of rHRP A2A and Enzyme Activity Assay

As obtained in our previous study, the rHRP A2A enzyme was purified in a single step using a 3-amino-4-chloro benzohydrazide affinity column [38]. Sodium dodecyl sulfate–polyacrylamide gel electrophoresis (SDS-PAGE) was performed to visualize the purified enzyme [39]. By measuring the absorbance increase brought on by the colored derivative compound (maximum: 420 nm) created by the oxidation of the o-dianisidine substrate in the presence of H_2_O_2_, the rHRP A2A enzyme activity was determined. The final reaction medium consisted of 0.8 mM o-dianisidine (200 μL), 60 mM phosphate buffer (pH 6.0) (600 μL), 2.25 mM H_2_O_2_ (100 μL), and 100 μL of enzyme. The activity (EU) was determined as the amount of enzyme catalyzing the formation of one µmol of derivative product per minute.

### Effects of Metal Ions on Enzyme Activity

To determine the effects of different metal ions on the activity of the pure rHRP A2A isoenzyme, the initial activity of the enzyme was determined using a fixed volume of enzyme (100 µL) [40, 41]. Compounds: CaCI_2_. 2H_2_O, FeCI_2_. 4H_2_O, FeCI_3_. 6H_2_O, MnSO_4_. H_2_O, and Cu(NO_3_)_2_. 5H_2_O were used in the studies. Solutions of the compounds were prepared with distilled water. Before examining the effect of metal ions on the enzyme, the absorbance of the solutions containing metal and substrate in the enzyme-free medium was checked against the blank in the spectrophotometer. This control was performed before all studies. Prior to studying each metal ion, the enzyme’s activity was determined using the provided method. Then, various metal solution concentrations (0–1 mM) were added to the reaction medium (0.8 mM o-dianisidine (200 µL), 60 mM phosphate buffer (pH 6.0) (600 µL), 2.25 mM H_2_O_2_ (100 µL), and 100 µL of enzyme) while maintaining the same amount of enzyme (100 µL), and by measuring the absorbances with a spectrophotometer, the enzyme activity was recalculated. The results were evaluated together with the initial enzyme activity. The absorbance of the metals was checked against the blank in a spectrophotometer, and no absorbance was detected. This check was done before all studies.

### Decolorization of Dyes with rHRP A2A 

The parameters affecting the degree of biodegradation of peroxidase-catalyzed dye are the pH of the reaction mixture, dye concentration, hydrogen peroxide concentration, enzyme concentration, and temperature. The optimal pH value was determined by monitoring the biodegradation rate between pH 4–7.5. For this purpose, 50 mM buffers at pH 4–5 (acetate buffer) and pH 6–7 (phosphate buffer) were prepared and used. To determine the effect of enzyme concentration on dye removal, biodegradation was varied between 0.004 and 0.04 EU/mL and monitored for 24 h for rHRP A2A. Absorbance was recorded at different time intervals. Then, under optimal pH and enzyme concentration, the effect of dye and hydrogen peroxide concentration on biodegradation was examined. Dyes were prepared at concentrations ranging from 10 to 100 mg/L, and their absorbance was recorded at the wavelength of each dye. Enzyme and H_2_O_2_ were added to the dye solution prepared at the determined concentrations, and the solution was incubated. Absorbances were recorded at different times. Thus, the dye concentrations with the highest removal were determined. Then, while holding the other variables constant, the effects of pH, H_2_O_2_ concentration, enzyme quantity, and temperature on dye removal were assessed for each parameter under study. Also, color removal studies were performed with a redox mediator on the dye with the highest removal according to the % decolorization results. For this, various amounts of 1 mM stock ABTS solution were added to the reaction, and the resulting concentrations were calculated and graphed. The ABTS concentration providing the highest color removal was determined. It was compared with the dye solution without ABTS.

The decolorization of dyes was calculated as % by the following equation.

$$\%Decolorization=\frac{Ai-At}{Ai}\times100$$

*Ai*: initial absorbance value of the dye;

*At*: absorbance value at the end of the specified time.

## Result and Discussion

### Production and Purification of rHRP A2A

Eukaryotic systems are utilized to produce recombinant HRP because native HRP has disulfide bridges and glycosylation. In 1992, hyperglycosylated HRP was produced using *Saccharomyces cerevisiae* [42]. Additionally, 19 different HRP isoenzymes and HRP mutants with higher activity and stability were produced in methylotrophic *K. phaffii* (*P. pastoris*) [43, 44]. In this study, the selected clone was grown in a BMMY medium with methanol added every 24 h for rHRP A2A isoenzyme expression in *K. phaffii* [45]*.* Methanol acts as an inducer and carbon source, while glycerol offers higher biomass yield but has repressive effects. Co-substrate glycerol increases biomass yield but inhibits the AOX1 promoter, reducing the amount of recombinant proteins produced [46–49]. The medium was supplemented with 1% (w/v) sorbitol as a carbon source and 1% (w/v) casamino acid, a hydrolysate of proteins, to reduce protease production [50].

**Fig. 2:**
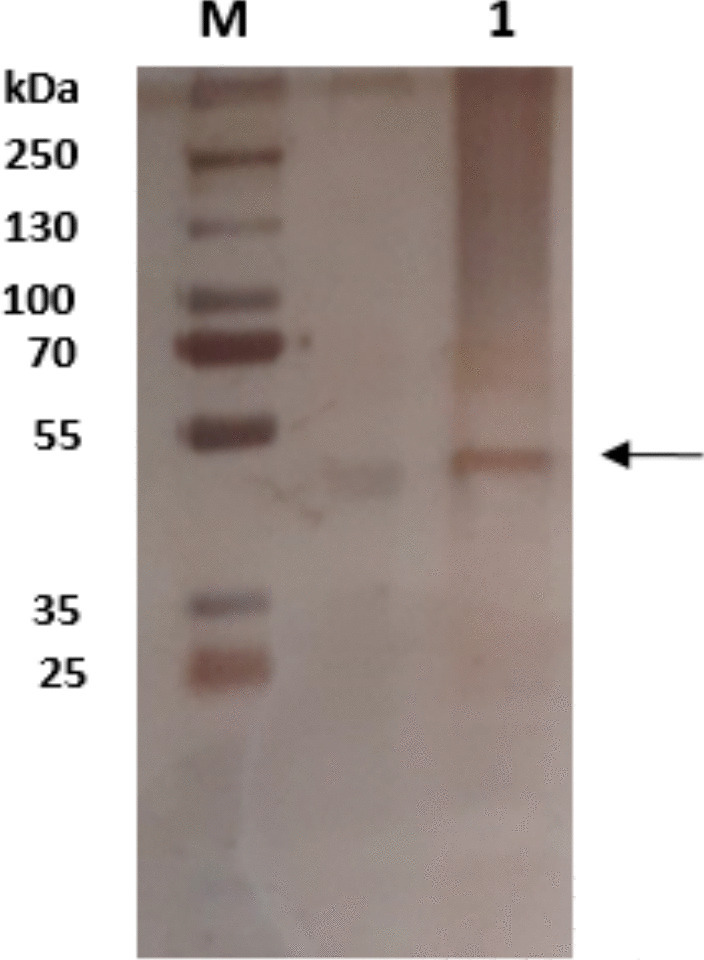
SDS-PAGE analysis of purified rHRP A2A (M: protein marker, 1: purified enzyme)

The horseradish plant’s root contains a combination of distinct isoenzymes, including the HRP enzyme, and it is exceedingly challenging to isolate these enzymes [51]. Therefore, there is a need for alternative methods that can be faster and cheaper, where the enzyme can be obtained and purified more easily. Recombinant production is very advantageous since it makes it possible to produce the desired isoenzyme. As reported in the previous study, rHRP A2A isoenzyme was produced extracellularly in *K. phaffii* cells and purified by affinity chromatography in a single step [38]. In this study, the recombinant enzyme produced and purified using the same method was demonstrated by silver staining after being run on SDS-PAGE (Fig. 2).

### The Effect of Metal Ions on the Activity of rHRP A2A

Peroxidases require different metal ions as their prosthetic groups or activators to perform their oxidative agent function [52–54]. In general, metal ions can coordinate with active site residues and lead to activation. Conversely, such coordination can also cause inhibition by blocking substrate interaction [55]. Recently, the effects of some metals on the functional stability of peroxidases have been reported [56]. The activation, inhibition, and denaturation effects of transition metals (Mn^2+^, Co^2+^, Ni^2+^, and Cu^2+^) on HRP in the presence of guaiacol as an aromatic donor were investigated. Depending on the applied concentration, they have been shown to affect horseradish peroxidase (HRP) activity. Such metal ions are strong inducers of the conformational and functional stability of HRP [57].

**Fig. 3:**
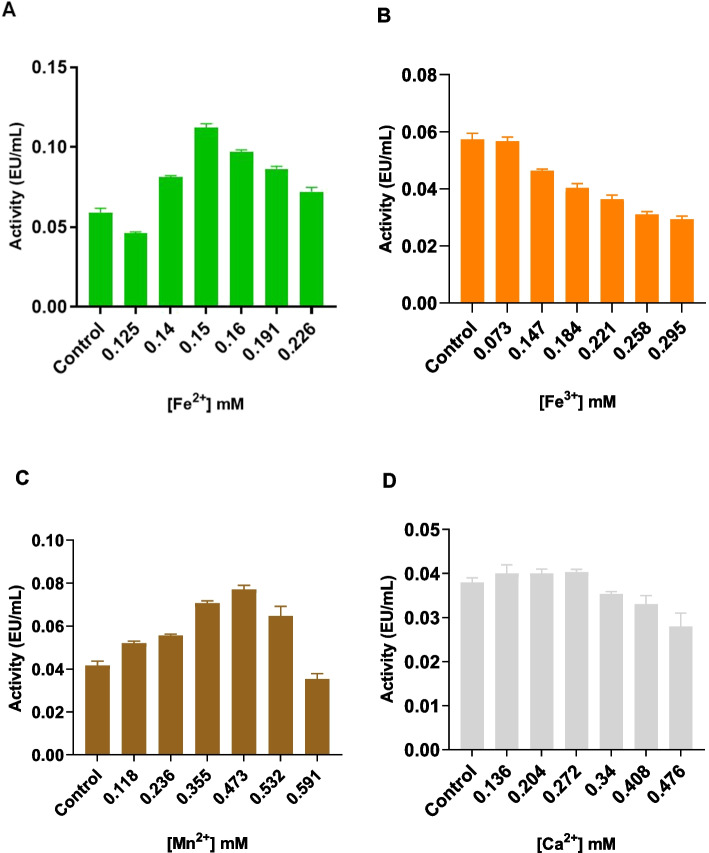
Effect of metal ions on rHRP A2A activity. Effect of **A** Fe^2+^, **B** Fe^3+^, **C** Mn^2+^, and **D** Ca^2+^. Control: without addition of metal ions

The effect of various metal ions (final concentration 0.2 mM) such as Cu^2+^, Fe^2+^, Ni^2+^, Hg^2+^, Ca^2+^, Mg^2+^, Zn^2+^, Mn^2+^, and K^+^ on *Citrus medica* leaf peroxidase enzyme activity was investigated. The enzyme activity was observed to rise in the presence of Cu^2+^, Co^2+^, Mg^2+^, and K^+^, but to drop to 84.33, 39.26, 62.33, and 63% of the initial activity in the presence of Hg^2+^, Fe^2+^, Mn^2+^, and Zn^2+^ [58]. In light of the literature, the effect of metal ions on pure rHRP A2A isoenzyme activity was investigated in this study. The activities of purified rHRP A2A isoenzyme were determined by adding salts containing Mn^2+^, Fe^2+^, Fe^3+^, and Ca^2+^ metal ions in the range of 0.03–0.6 mM. The activity of the control group (without addition of metal ions) was compared (Fig. 3). Peroxidases perform oxidation–reduction reactions with iron ions in their active center [59]. Iron is considered essential for the activities of plant peroxidases because it plays a role in H_2_O_2_ binding and the formation of compound I [60]. The effects of chloride salts of metal ions Ca^2+^, Mn^2+^, and Fe^3+^ at different concentrations on the enzyme were investigated by Goyal and Chugh. It was stated that the addition of Fe^3+^ increased the activity of peroxidase isolated from pearl millet grains, but Mn^2+^ moderately inhibited the enzyme activity [61]. There is also evidence for the inhibitory effects of Mn^2+^ (MnCI_2_) on peroxidase isolated from cotton cell suspension [62] and black lentil peels [63].

The data showed that the activity of pure rHRP A2A isoenzyme increased by 158.62 and 79.54%, respectively, when 0.150 mM Fe^2+^ and 0.473 mM Mn^2+^ were added to the activity measurement medium. The results revealed that the Fe^2+^ ion is an important factor in the activity of the rHRP A2A isoenzyme.

Ca^2+^ is a cofactor that helps to maintain the conformational integrity of the active site of the peroxidase enzyme [64]. Avocado, cereal, and wheat germ peroxidase enzyme activities have been reported to be activated by Ca^2+^ [65, 66]. Onsa et al. reported that the activities of mPOD isoenzymes isolated from *Metroxylon sagu* were variously affected by the presence of metal ions. It was determined that 1 mM Fe^3+^ significantly increased the activity of both mPOD-I (339%) and mPOD-II (328%) [67]. However, while it was observed that Fe3 + ion caused inhibition, the enzyme activity remained stable in a certain concentration range of Ca^2+^ ions. This could imply that metals do not have the same effect on the activities of various source peroxidases and isoenzymes. It was observed that the Cu^2+^-containing compound interacted with the substrate and formed a precipitate, and it was determined that the formed particles caused an increase in absorbance. The impact of Cu^2+^ ions on enzyme activity could not therefore be investigated.

### Decolorization of Dyes with rHRP A2A

Isolated enzymes are highly efficient and considered environmentally friendly, as they are highly specific catalysts that produce by-products with lower toxicity and volume. The enzymes responsible for dye degradation mainly belong to the family of oxidoreductases, including peroxidases, reductases, and laccases [68, 69]. Horseradish peroxidase (HRP), the most important representative of peroxidases, is the most studied enzyme derived from plant material, used as a very effective biocatalyst for the treatment of various recalcitrant pollutants (i.e., dyes, phenols) in wastewater [70–74].

**Fig. 4:**
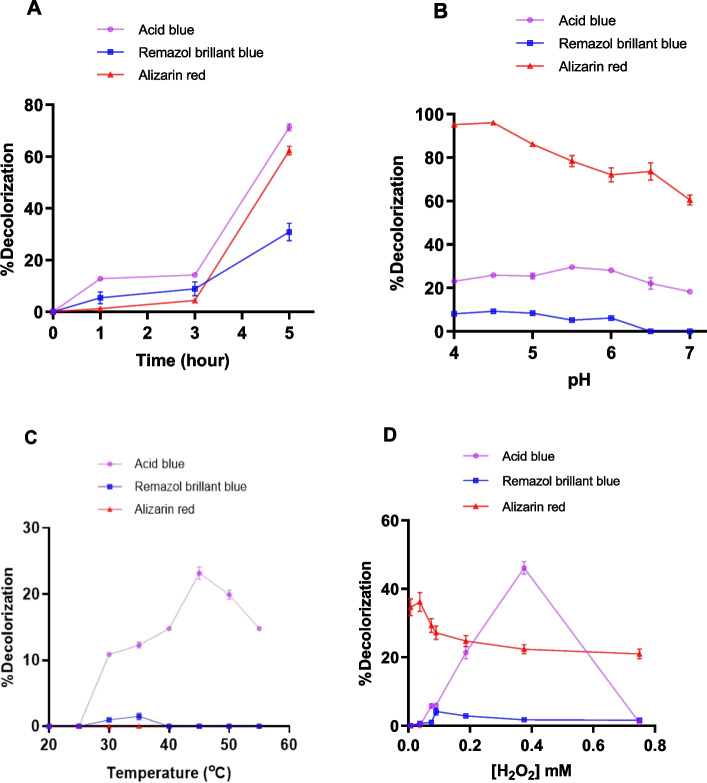
**A** The change of dye decolorization with time. **B** Effect of pH. **C**. Effect of temperature. **D**. Effect of H_2_O_2_ concentration

Optimization conditions vary in enzymatic dye degradation studies. Souza et al. studied the decolorization of different dyes and observed that the reaction time is directly dependent on the different structures of the dyes, resulting in variation in the reaction time [75]. In the present study, the decolorization potential of pure rHRP A2A isoenzyme on one azo dye (Acid blue 113) and two anthraquinone dyes (Alizarin red and Remazol brillant blue), which are widely used in the textile field, was investigated, and optimization studies were carried out. The % decolorization rates of Acid blue 113, Remazol brilliant blue, and Alizarin red dyes with a concentration of 10 mg/L at the end of 1, 3, and 5 h were calculated and graphed. The graphs show that for all three dyes, the greatest amount of decolorization occurred after the 5 th hour. The dye with the best color decolorization was Acid blue 113 (71.27%), followed by Alizarin red (62.26%) and Remazol brilliant blue (31.22%) (Fig. 4A). According to the % decolorization values obtained with the buffers used in the pH 4–7 range to determine the effect of pH on the color decolorization of the dyes studied, for Acid blue 113, the maximum decolorization was attained at pH 6.0, whereas Remazol brilliant blue and Alizarin red dyes accomplished this at pH 4.5 (Fig. 4B). Enzymes isolated from different sources have also different optimum pHs. *Trichosanthes dioica* peroxidase functioned better in acidic conditions with a pH range of 3–5, whereas its decolorizing/degrading activity was negatively affected in alkaline conditions [76]. Degradation of industrially important dyes by enzymes such as horseradish peroxidase, polyphenol oxidase, bitter melon peroxidase, and laccase is maximal in acidic pH buffers [77]. In decolorization experiments with HRP, a decrease in the activity of HRP was observed at pH values above 6.0 [78]. The optimum pH for rHRP A2A enzyme activity was determined as 6.0 in our previous study [38]. In the literature, to determine the HRP-catalyzed degradation of Remazol brilliant blue, the absorbance decrease of 15 mg/L dye was determined at various pH values (3.0–8.0) with plant-derived HRP enzyme (0.14 EU/mL) [78]. For Remazol brilliant blue, the HRP enzyme showed much better dye decolorization at acidic pH than at neutral pH [79].

To determine the effect of temperature on dye removal, dye absorbance changes between 20 and 55 °C were determined, and % decolorization values were calculated. The reaction temperature is an important parameter affecting the color removal of dyes. In the literature, it was determined that the optimal temperature varies according to the structure of the dye in the decolorization studies carried out with HRP [36]. It is believed that the decrease in dye removal at high temperatures may be linked to the loss of enzymatic activity [80–82]. According to the obtained data, the maximum % decolorization of Acid blue 113 was reached at 45 °C. The best decolorization for Remazol brilliant blue was determined at 25 °C. No effect of temperature on decolorization for Alizarin red dye was observed (Fig. 4C). As seen in the graph, besides the different effects of temperature on each dye, the decrease in dye removal at increasing temperatures suggests that it may be related to the deactivation of the enzyme.

To determine the effect of H_2_O_2_ on dye decolorization, H_2_O_2_ was added to the reaction medium in the range of 0.0075–0.75 mM. Absorbances were determined spectrophotometrically, and % decolorization was calculated. As seen in the graph, maximum decolorization of Acid blue 113 was observed in the presence of 0.375 mM H_2_O_2_. As for Remazol brilliant blue and Alizarin red, the highest % decolorization was observed in the presence of 0.09 mM and 0.0375 mM H_2_O_2_, respectively (Fig. 4D). The sensitivity of peroxidases to high concentrations of H_2_O_2_ has also been reported in previous studies [30, 31, 82–85]. It has also been observed that low concentrations of H_2_O_2_ inhibit enzyme action, and excess of this reagent causes enzyme inactivation [86]. According to the results of this study, the concentration of H_2_O_2_ required for maximum oxidation of each dye has shown changes. The difference in the level of oxidation between dyes with various structures explains this. In addition, decolorization percentages decreased as the H_2_O_2_ concentration increased. This indicates that high H_2_O_2_ inhibits the enzyme as stated in the literature.

**Fig. 5:**
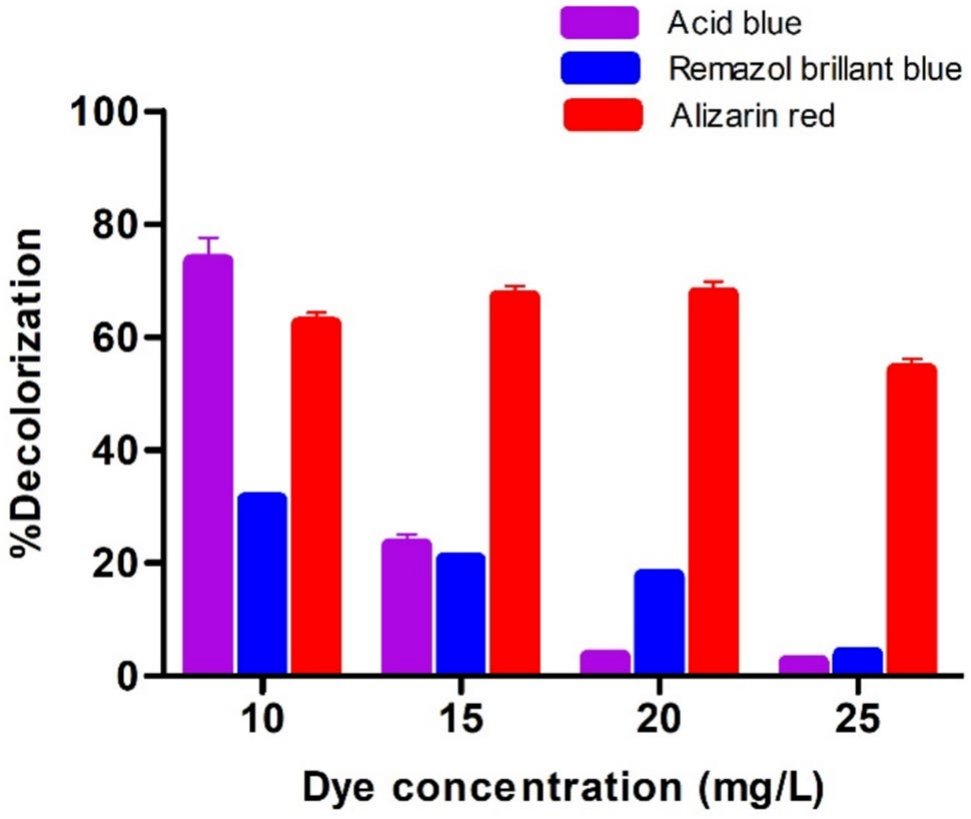
Graph of % decolorization at different dye concentrations

In the decolorization studies performed at different dye concentrations (10–100 mg/L), the maximum % decolorization of Acid blue 113, Alizarin red, and Remazol brilliant blue for 10 mg/L dye was 71.27, 62.26, and 31.22%, respectively. No removal was observed at dye concentrations above 25 mg/L (Fig. 5).

**Fig. 6:**
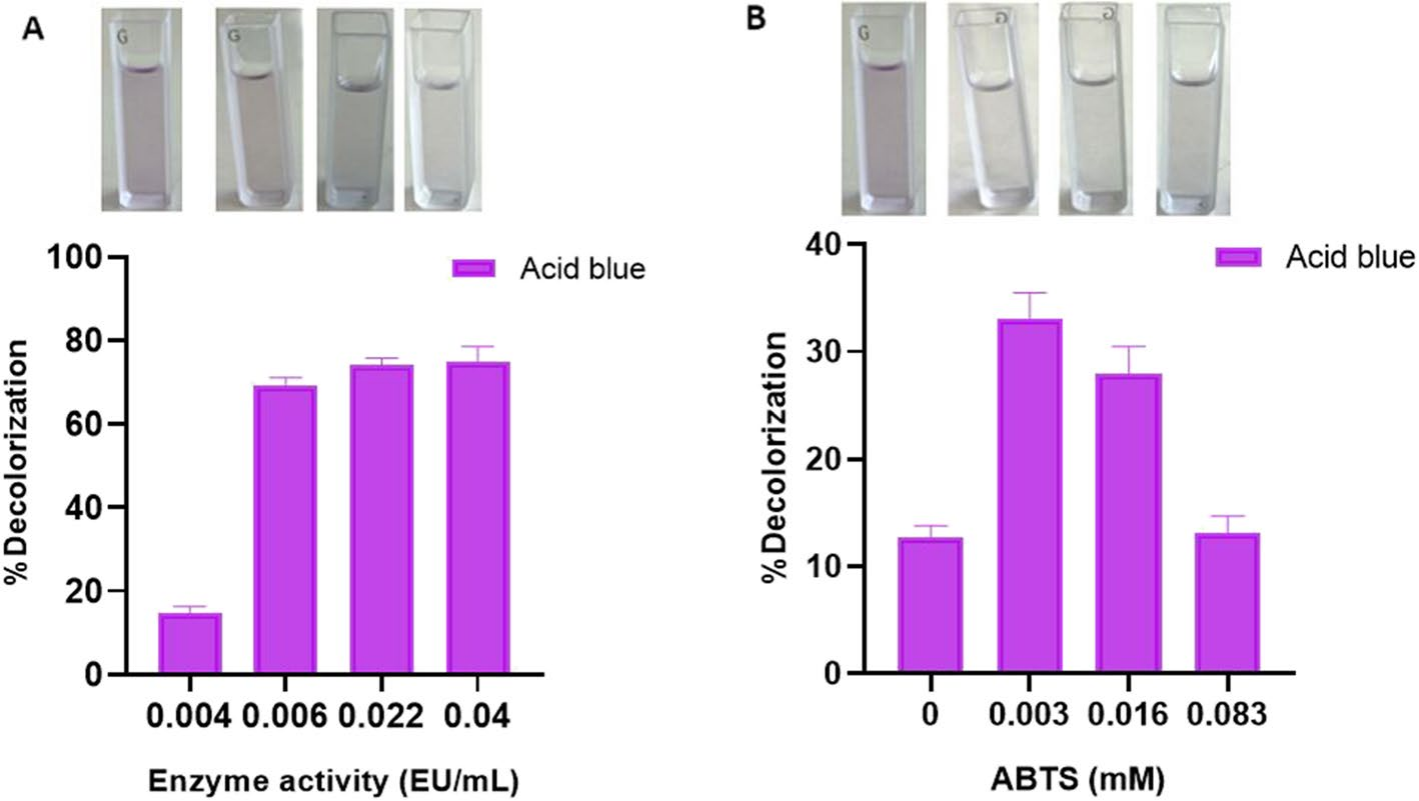
**A** Enzyme activity (EU/mL)-%decolorization graph. **B** ABTS (mM)-% decolorization graph

It was also aimed to increase the color removal by increasing the enzyme activity for Acid blue 113 dye, which was best removed with 71.27% decolorization for 10 mg/L dye concentration. For this purpose, dye decolorization was studied using rHRP A2A with increasing activity in the range of 0.004–0.04 EU/mL given in Fig. 6A. While 71.27% decolorization was obtained in 5 h with 0.006 EU/mL activity, 74.83% dye % decolorization was achieved when the activity was increased to 0.04 EU/mL. Consequently, it was determined that dye removal increased as the enzyme activity increased. The enzyme activity also increased with the addition of metal ions. Thus, it enabled dye removal studies to be carried out using less amount (mL) of the enzyme. Sadhanandam et al. used 0.08 EU/mL plant-derived HRP enzyme and degraded 30 mg/L acid blue 113 dye up to 75% in 45 min at pH 6.6 and 30 °C [87]. Compared to this study, three times more dye can be removed by using 13 times more active enzymes with about 75%, while 71.27% decolorization of Acid blue 113 dye was achieved with rHRP A2A with an activity of 0.006 EU/mL. This indicates that even at low enzyme activity, high decolorization rates can be attained.

Additionally, for the Acid blue 113 dye with the best removal, % decolorization values were calculated and graphed by adding different concentrations of ABTS to the medium. According to Husain (2006), enzymes alone cannot degrade some recalcitrant dyes, and therefore, they need some low molecular weight redox mediators to increase the dye degradation efficiency. Redox mediators speed up the degradation process by moving electrons from biological electron donors to electron acceptor dye compounds [88]. A redox mediator such as ABTS is known to increase substrate diversity and accelerate the decolorization efficiency of resistant textile dyes [89]. In this context, the decolorization of Acid blue 113 dye was examined by adding ABTS at different concentrations. According to the results, in the non-ABTS % decolorization study, 12.74% removal was detected after 3 h, while 33.123% decolorization was achieved after 2 h with the addition of 0.003 mM ABTS (Fig. 6B). Thus, it was demonstrated that the presence of a redox mediator at the appropriate concentration provides dye decolorization in a shorter time and at a higher rate.

## Conclusion

This study highlights the potential of the rHRP A2A enzyme as an effective biological agent for the decolorization of toxic dyes Acid blue 113, Alizarin red, and Remazol brilliant blue R. While numerous physical and chemical methods have been proposed for the decolorization of synthetic dyes, these approaches often have drawbacks, including high costs, low efficiency, and energy-intensive processes. In contrast, biodegradation of dyes using enzymes is recognized as a cost-effective and environmentally friendly option. In this study, the azo and anthraquinone dye removal potential of the rHRP A2A enzyme, which was produced extracellularly by recombinant *K. phaffii* cells and purified in a single step by the affinity chromatography technique using the 3-amino-4-chloro benzohydrazide ligand, was demonstrated. The optimum conditions for maximum dye degradation, including time, pH, temperature, dye concentration, and substrate concentration, were determined. Under these optimal conditions, the highest percentage of decolorization was achieved for Acid blue 113 dye, with a removal rate of 71.27%. This was followed by Alizarin red at 62.26% and Remazol brilliant blue R at 31.22%. These results indicate that rHRP A2A has significant decolorization capabilities, particularly for Acid blue 113 and Alizarin red dyes, highlighting its potential for treating textile wastewater.

## Data Availability

Data will be made available on reasonable request.
